# Beyond Menopausal Stage: Symptom Burden, Self-Efficacy, and Psychological Distress

**DOI:** 10.3390/jcm15186944

**Published:** 2026-09-08

**Authors:** Andrei Pescaru, Flavia Crișan, Alexia Manole, Daiana Cerasela Ivanis, Camelia Corina Pescaru, Victor Buciu, Cristina Secosan, Adelina Marițescu, Bogdan Adrian Manta, Roxana Folescu

**Affiliations:** 1 Doctoral School, “Victor Babes” University of Medicine and Pharmacy Timisoara, 300041 Timisoara, Romaniavictor.buciu@umft.ro (V.B.); 2 Research Centre for Pharmaco-Toxicological Evaluations, University Clinic of Toxicology, Pharmaceutical Industry, Management and Legislation, Faculty of Pharmacy, ‘Victor Babeș’ University of Medicine and Pharmacy Timișoara, 300041 Timisoara, Romania; 3 Faculty of Medicine and Pharmacy, University of Oradea, 410087 Oradea, Romania; manole.alexia@student.uoradea.ro; 4 Department of Neonatology and Puericulture, “Louis Ţurcanu” Children Emergency Hospital, 300011 Timisoara, Romania; ivanis_daiana@yahoo.com; 5 Center for Research and Innovation in Personalized Medicine of Respiratory Diseases, “Victor Babes” University of Medicine and Pharmacy Timisoara, 300041 Timisoara, Romania; pescaru.camelia@umft.ro; 6 Department of Obstetrics and Gynecology, “Victor Babes” University of Medicine and Pharmacy Timisoara, 300041 Timisoara, Romania; secosan.cristina@umft.ro; 7 Department of Pulmonology, Faculty of Medicine, “Victor Babes” University of Medicine and Pharmacy Timisoara, 300041 Timisoara, Romania; adelina.maritescu@umft.ro; 8 Department I—Nursing, Clinical Skills University Clinic, Faculty of Nursing, “Victor Babes” University of Medicine and Pharmacy Timisoara, 300041 Timisoara, Romania; bogdan.manta@umft.ro; 9 Department of Balneology, Medical Recovery and Rheumatology, Family Medicine University Clinic, Center for Preventive Medicine, Center for Advanced Research in Cardiovascular Pathology and Hemostaseology, “Victor Babes” University of Medicine and Pharmacy Timisoara, 300041 Timisoara, Romania; folescu.roxana@umft.ro

**Keywords:** menopause, self-efficacy, psychological distress

## Abstract

**Background:** Psychological distress during menopause may reflect the combined influence of symptom burden and psychological resources. This study examined the concurrent conditional associations of nonpsychological menopausal symptom burden and general self-efficacy with anxiety and depressive symptom severity. **Methods:** This cross-sectional study included 247 peri- and postmenopausal women recruited from hospital and community settings in Timișoara, Romania. Participants completed the Hospital Anxiety and Depression Scale, Menopause Rating Scale, Menopause-Specific Quality of Life Questionnaire, General Self-Efficacy Scale, and COPE Inventory. Associations were examined using Spearman correlations and multivariable linear regression with HC3 robust inference. **Results:** At least borderline anxiety and depressive symptoms were identified in 54.7% and 42.5% of participants, respectively. In the fully adjusted psychological-resource models, greater nonpsychological menopausal symptom burden was associated with higher anxiety (β = 0.311, *p* < 0.001) and depression scores (β = 0.239, *p* < 0.001). Higher general self-efficacy was associated with lower anxiety (β = −0.169, *p* = 0.009) and depression scores (β = −0.319, *p* < 0.001). Although self-reported surgical menopause was associated with lower anxiety and depression scores relative to early perimenopause in the primary models, these associations were attenuated and no longer statistically significant in the restricted sensitivity analysis. **Conclusions:** Nonpsychological menopausal symptom burden and general self-efficacy were concurrent conditional correlates of anxiety and depressive symptom severity. The cross-sectional design does not establish temporal direction or causal effects, and the surgical-menopause finding should not be interpreted as protective.

## 1. Introduction

Menopause is a natural part of reproductive aging, while the menopausal transition is a diverse stage marked by changes in menstrual cycles and ovarian activity [1]. Throughout this period, women may encounter vasomotor, somatic, urogenital, sleep-related, and psychological symptoms, which differ widely in occurrence and intensity. These symptoms can affect daily life and menopause-related quality of life, highlighting the importance of identifying factors linked to psychological distress as a key aspect of thorough menopause management.

Anxiety and depression are especially relevant during the menopausal transition. Evidence from the Study of Women’s Health Across the Nation shows that women are two to four times more likely to have a major depressive episode during perimenopause or early postmenopause compared to premenopause, regardless of previous depression, stress, vasomotor symptoms, psychotropic medication use, or hormone levels [2]. Recent SWAN studies reveal that depressive-symptom patterns around the final menstrual period vary and are affected by anxiety, stress, sleep issues, and physical health [3]. Anxiety symptoms can also predict later major depression in midlife women [4]. These results imply that psychological outcomes during menopause are influenced by more than just the menopausal stage and should be considered within a broader biopsychosocial context.

Menopausal symptom burden is strongly linked to psychological well-being. Cross-sectional studies have found positive correlations between the severity of menopausal symptoms and levels of anxiety and depression [5]. Additionally, tools like the Menopause Rating Scale and the Menopause-Specific Quality of Life Questionnaire can identify women with higher anxiety and depression scores [6]. However, since psychological items in menopause symptom scales may overlap with measures of anxiety and depression, focusing on nonpsychological menopausal symptoms—such as somatic, vasomotor, and urogenital complaints—could offer a more cautious assessment of their concurrent association with psychological distress.

General self-efficacy, defined as an individual’s belief in their ability to manage difficult situations and achieve desired outcomes, has been examined as a psychological resource in relation to menopausal well-being. Previous studies have reported inverse associations of self-efficacy and resilience with psychological complaints across menopausal stages [7] and a statistical interaction between self-efficacy and menopausal symptom severity in relation to life satisfaction [8]. These findings support examining self-efficacy as a concurrent psychological correlate, but they do not establish whether self-efficacy precedes psychological distress. Depressive or anxiety symptoms may also reduce perceived competence, while both self-efficacy and psychological distress may reflect shared underlying factors such as perceived control or demoralization. Coping strategies have likewise been examined in relation to psychological adjustment, although the relationships among specific coping responses, menopausal symptom burden, and psychological outcomes have varied across studies [9].

Previous studies have generally examined menopausal symptoms, quality of life, self-efficacy, or coping strategies separately. Evidence remains limited regarding their concurrent conditional associations with anxiety and depressive symptom severity after accounting for menopausal stage and relevant sociodemographic and clinical characteristics. Context-specific evidence concerning these relationships among Romanian peri- and postmenopausal women is also limited. Examining these factors together may help characterize clinically relevant patterns and generate hypotheses for longitudinal research; however, cross-sectional associations cannot establish whether self-efficacy is modifiable, whether it precedes psychological distress, or whether interventions targeting self-efficacy would improve psychological outcomes.

Therefore, the objective of this cross-sectional study was to examine the concurrent conditional associations of nonpsychological menopausal symptom burden and general self-efficacy with anxiety and depressive symptom severity among Romanian peri- and postmenopausal women. Secondary objectives were to explore the relationships of coping strategies and menopausal stage with the two psychological outcomes and to assess the robustness of the principal associations across alternative model specifications. Given the cross-sectional design, the analyses were not intended to identify causal effects, mediation pathways, or the temporal direction of the observed associations.

## 2. Materials and Methods

### 2.1. Study Design and Setting

This cross-sectional observational study was conducted in Timișoara, Romania, from 27 February to 31 July 2026. Participants were recruited via two methods: hospital-based recruitment involved current or former patients of the Department of Obstetrics and Gynecology at the Municipal Emergency Clinical Hospital of Timișoara, while community recruitment was conducted outside the hospital through community contacts and personal networks. These dual approaches aimed to include women with varying degrees of engagement with healthcare services.

Data were collected in a single session using a structured, self-administered Romanian questionnaire that covered sociodemographic details, clinical information, and standardized patient-reported outcome measures. Medication exposure was self-reported and coded as two binary variables: reported use of menopause-related medication and reported use of an SSRI or an anxiolytic. The questionnaire did not record the specific medication, therapeutic indication, dose, treatment duration, adherence, or treatment response. These variables were therefore included only as covariates in the extended sensitivity models and were not used to evaluate medication effectiveness. There was no intervention or follow-up assessment. The study was designed and reported in accordance with the STROBE guidelines for cross-sectional studies [10].

The Ethics Committee of the Municipal Emergency Clinical Hospital of Timișoara approved the study protocol on 27 February 2026 (approval No. E-891/27.02.2026). All participants gave written informed consent prior to enrollment. The study adhered to the principles of the Declaration of Helsinki [11].

### 2.2. Conceptual Framework

The conceptual framework used to organize covariate selection is presented in Supplementary Figure S1. The directed acyclic graph represents hypothesized relationships in which measured sociodemographic and clinical factors may be related to nonpsychological menopausal symptom burden, general self-efficacy, and psychological distress, while nonpsychological symptom burden and general self-efficacy were treated as the focal concurrent correlates of the psychological outcomes. The arrows were used to organize the analytical framework and should not be interpreted as causal effects established by the present cross-sectional data. Because all constructs were measured concurrently, reverse directionality among psychological distress, symptom reporting, and perceived general self-efficacy remains possible.

The conceptual framework organized the analyses rather than claiming that covariate adjustment identified causal effects. Nonpsychological menopausal symptom burden was treated as the focal correlate in the symptom-burden model, and general self-efficacy was added in the psychological-resource model. We included age, body mass index, menopausal stage, recruitment source, education, employment status, marital status, and comorbidity to improve comparability across measured participant characteristics. We examined medication variables in sensitivity analyses because we could not establish their timing or treatment indication. We analyzed coping strategies separately as exploratory concurrent correlates rather than as established confounders or mediators.

### 2.3. Participants

Women were eligible if they were at least 40 years old, were in early or late perimenopause, natural postmenopause, or self-reported surgical menopause, were able to understand and complete the questionnaires in Romanian, and provided written informed consent. The natural menopausal stage was determined from self-reported menstrual history using the STRAW+10 criteria. Early perimenopause was defined by a persistent difference of at least seven days in the length of consecutive menstrual cycles, late perimenopause by an interval of amenorrhea lasting at least 60 days but less than 12 months, and natural postmenopause by amenorrhea lasting at least 12 months. Participants who reported menopause following gynecological surgery involving removal of the ovaries and/or uterus were classified in a separate self-reported surgical-menopause category. The questionnaire did not distinguish hysterectomy alone from unilateral or bilateral oophorectomy and did not collect the indication for surgery, the exact procedure, or the date of surgery; therefore, this category should not be interpreted as a clinically verified bilateral-oophorectomy group.

Women were excluded if they were younger than 40 years, declined to participate, had cognitive or communication difficulties that prevented questionnaire completion, or had primary-outcome scores that could not be verified from the original item-level responses. A total of 270 women completed the study questionnaire. For 23 participants, entered HADS subscale totals were available, but the corresponding original item-level responses could not be retrieved in a form that allowed independent verification of scoring. Because HADS-A and HADS-D were the primary outcomes, we excluded these 23 participants from the outcome analyses, leaving 247 women in the analytical sample. Available non-HADS data from the 23 excluded participants were retained only for a comparison of included and excluded participants and were not used to reconstruct or impute the primary outcomes.

### 2.4. Hospital Anxiety and Depression Scale

Anxiety and depressive symptoms were measured with the Hospital Anxiety and Depression Scale (HADS) [12]. The scale has 14 items: seven for anxiety (HADS-A) and seven for depression (HADS-D). Each item is scored from 0 to 3, resulting in subscale scores ranging from 0 to 21. Higher scores reflect more severe symptoms. For description, scores were classified as normal (0–7), borderline (8–10), or clinical range (11–21). A score of 8 or above suggests at least borderline symptoms. HADS categories served for screening and descriptive purposes, not as psychiatric diagnoses. The continuous scores of HADS-A and HADS-D were used in correlation and regression analyses.

### 2.5. Menopause Rating Scale

Menopausal symptom burden was measured using the 11-item Menopause Rating Scale (MRS), which comprises somatic, psychological, and urogenital domains [13]. Each item is scored from 0 (no symptoms) to 4 (very severe symptoms). The somatic domain includes hot flushes and sweating, heart discomfort, sleep problems, and joint and muscular discomfort; the psychological domain includes depressive mood, irritability, anxiety, and physical and mental exhaustion; and the urogenital domain includes sexual problems, bladder problems, and vaginal dryness. The total MRS score ranges from 0 to 44, while the somatic, psychological, and urogenital domain scores range from 0 to 16, 0 to 16, and 0 to 12, respectively. For the principal analyses, a seven-item nonpsychological MRS score was calculated by summing the four somatic and three urogenital items, yielding a possible range of 0 to 28. The psychological domain, including the physical and mental exhaustion item, was therefore excluded from this composite to reduce direct item-content overlap with the HADS outcomes. The psychometric characteristics of the seven-item nonpsychological MRS composite were examined in all participants. Internal consistency was assessed using Cronbach’s alpha. The structure of the composite was explored using principal component analysis of the item correlation matrix, and the relationship between the somatic and urogenital domain scores was assessed using Spearman’s rank correlation. Because the composite intentionally combines two clinically distinct MRS domains, these analyses were used to describe its internal structure rather than to assume strict unidimensionality.

### 2.6. Menopause-Specific Quality of Life Questionnaire

Menopause-specific quality of life was evaluated with the 29-item Menopause-Specific Quality of Life Questionnaire (MENQOL) [14], which assesses four areas: vasomotor, psychosocial, physical, and sexual functioning. Participants indicated whether each symptom occurred in the past month. Symptoms not present scored 1. For symptoms that did occur, the level of bother was rated from 0 to 6 and then transformed into a score from 2 to 8. Domain scores were computed as the average of the converted item scores within each domain, while the overall MENQOL score was the mean of all 29 items. Consequently, all scores range from 1 to 8, with higher scores reflecting poorer menopause-specific quality of life. In the present study, MENQOL total score was treated as a descriptive and exploratory correlate of psychological distress rather than as a focal exposure or adjustment variable. MENQOL includes psychosocial, physical, vasomotor, and sexual content that overlaps conceptually with both menopausal symptom burden and psychological functioning and may represent the overall impact or consequence of these experiences. It was therefore not included in the primary multivariable models to avoid construct redundancy and potential overadjustment; no causal or mediating role was assigned to MENQOL.

#### General Self-Efficacy Scale

General self-efficacy was measured with the 10-item General Self-Efficacy Scale (GSES) [15]. Each item is rated on a 1 to 4 scale, with scores summed to yield a total between 10 and 40. No items are reverse-scored. Higher total scores reflect a greater perceived ability to handle challenging or unexpected situations.

### 2.7. COPE Inventory

Coping strategies were evaluated using the Romanian version of the 60-item Coping Orientation to Problems Experienced (COPE) Inventory [16,17]. This tool measures 15 coping strategies, each with four items. Responses are rated from 1 to 4, with higher scores reflecting more frequent use of that strategy. Subscale scores were computed as the mean of their four items, resulting in scores from 1 to 4.

For clarity, coping subscales were grouped following the predefined database scoring scheme. Problem-focused coping included active coping, restraint, suppression of competing activities, and planning. Emotion-focused coping encompassed positive reinterpretation, instrumental support, religion, emotional support, and acceptance. Avoidant coping involved mental disengagement, venting, denial, behavioral disengagement, and substance use. Humor was retained as a separate COPE subscale but was not included in these broader groups.

The original analysis highlighted positive reinterpretation, planning, denial, and behavioral disengagement; however, this selection was not established through a formal preregistration and is therefore not described as prespecified in the revised manuscript. All 15 COPE subscales were examined as exploratory psychological correlates. The complete correlation profile was evaluated with false-discovery-rate correction, and a supplementary multivariable analysis entered all 15 subscales simultaneously to assess whether any coping strategy retained a conditional association with the psychological outcomes after adjustment. Coping variables were not included in the primary symptom-burden or psychological-resource models.

The initial sample-size calculation was based on detecting a bivariate correlation coefficient of 0.20 with 80% power at a two-sided significance level of 0.05, resulting in a minimum requirement of 194 participants. All eligible participants with verifiable primary-outcome data were included, yielding an analytical sample of 247 women. This calculation was not designed specifically for the final multivariable regression models and should not be interpreted as demonstrating adequate power for every adjusted coefficient or menopausal-stage comparison. Accordingly, model adequacy was not inferred from an observations-per-parameter ratio, and the precision of the principal estimates is presented using 95% confidence intervals.

### 2.8. Statistical Analysis

Statistical analyses were performed using MedCalc Statistical Software, version 23.4.0 (MedCalc Software Ltd., Ostend, Belgium). The distributions of continuous variables were evaluated with the Shapiro–Wilk test and visual inspection. Since most questionnaire scores did not follow a normal distribution, continuous variables are shown as medians with interquartile ranges, whereas categorical variables are listed as frequencies and percentages.

To evaluate potential selection associated with the exclusion of participants whose HADS scores could not be verified, the 247 included and 23 excluded participants were compared using all available non-HADS variables; continuous variables were compared using the Mann–Whitney U test, and categorical variables using Pearson’s chi-square test or the Fisher–Freeman–Halton exact test, as appropriate.

Spearman’s rank correlation coefficients (ρ) with 95% confidence intervals were computed for the relationships of HADS-A and HADS-D scores with psychological and nonpsychological MRS scores, MENQOL total score, GSES total score, and all 15 COPE subscales. These correlations were treated as exploratory. Benjamini–Hochberg false-discovery-rate correction was applied separately for each psychological outcome across the complete family of 19 correlation tests, and both nominal *p*-values and adjusted q-values were reported. In a supplementary multivariable coping analysis, all 15 COPE subscales were entered simultaneously together with nonpsychological MRS score, GSES score, and the measured sociodemographic and clinical covariates. False-discovery-rate correction was also applied across the 15 coping coefficients separately for each outcome.

For each psychological outcome, two nested multivariable linear regression models were constructed. The symptom-burden model included nonpsychological MRS score together with age, body mass index, menopausal stage, recruitment source, education, employment status, marital status, and comorbidity. The psychological-resource model added GSES total score to the symptom-burden model. This sequence was used to distinguish the focal symptom-burden and psychological-resource components and was not interpreted as a mediation analysis. Coping variables were excluded from the primary models and examined separately as exploratory correlates. All variables were entered simultaneously, and no stepwise selection was used.

An extended-covariate sensitivity model additionally included residence, smoking status, menopause-related medication use, and SSRI or anxiolytic use. Menopausal stage was entered as a four-category variable comprising early perimenopause, late perimenopause, natural postmenopause, and self-reported surgical menopause, with early perimenopause as the reference category. Community recruitment, non-university education, not employed, single/divorced/widowed, no comorbidity, rural residence, never smoking, and no medication use served as the respective reference categories. Results are expressed as unstandardized coefficients (B), standardized coefficients (β), HC3 heteroscedasticity-consistent 95% confidence intervals, and two-sided p-values. To formally compare the strength of the adjusted association between GSES and the two psychological outcomes, the difference between the standardized GSES coefficients for HADS-D and HADS-A (βHADS-D − βHADS-A) was estimated using a paired nonparametric bootstrap with 10,000 participant-level resamples; both outcome models were refitted within each resample, and a two-sided bootstrap confidence interval and *p*-value were calculated for the coefficient difference. As an exploratory effect-modification analysis, a product term between the mean-centered nonpsychological MRS and GSES scores was added to each fully adjusted psychological-resource model, while retaining the corresponding main effects and all model covariates; HC3 robust inference was used.

Multicollinearity was checked using variance inflation factors. Model assumptions and fit were assessed with residual-versus-fitted and Q–Q plots. Residual normality was assessed using the Shapiro–Wilk test, heteroscedasticity using the Breusch–Pagan test, and model form using the Ramsey regression specification error test. Influential points were identified using residuals, leverage values, and Cook’s distances. HC3 robust standard errors were applied to minimize the effects of heteroscedasticity and residual deviations from normality.

Model-specification sensitivity analyses evaluated functional form and outcome distribution. Nonpsychological MRS score, GSES score, and age were modeled using restricted cubic splines with four knots placed at the 5th, 35th, 65th, and 95th percentiles of their observed distributions. Joint Wald tests of the nonlinear spline terms were used to assess departure from linearity. Proportional-odds ordinal logistic regression models were also fitted using the established HADS categories of normal, borderline, and clinical-range symptoms. These ordinal models included the same predictors as the corresponding psychological-resource models.

Sensitivity analyses were performed to examine the composition of the nonpsychological MRS association. First, the somatic and urogenital domain scores were entered separately and then simultaneously in otherwise identical psychological-resource models using the full analytical sample (*N* = 247). Second, the nonpsychological score was recalculated after excluding the sleep-problems item, and the models were re-estimated using this six-item score. The seven constituent nonpsychological MRS items were also examined in separate adjusted models, with Benjamini–Hochberg false-discovery-rate correction applied across the seven item-level tests for each psychological outcome. The physical and mental exhaustion item was not included in these analyses because it belongs to the psychological MRS domain and was excluded from the original nonpsychological composite.

A sensitivity analysis was conducted to examine the self-reported surgical-menopause finding. This analysis was restricted to participants with natural postmenopause or self-reported surgical menopause who had available data for years since menopause (n = 147). Natural postmenopause was used as the reference category, and the surgical-menopause indicator and years since menopause were entered together with nonpsychological MRS score, GSES score, age, body mass index, recruitment source, education, employment status, marital status, comorbidity, menopause-related medication use, and SSRI or anxiolytic use. Indication for surgery, exact surgical procedure, and detailed hormone-therapy regimens were not available and could not be included.

No weighting or sampling-design analyses were performed; the only interaction analysis was the exploratory mean-centered MRS × GSES analysis described above. All tests were two-sided, and *p*-values below 0.05 were considered statistically significant.

## 3. Results

Participant characteristics are summarized in Table 1. Natural postmenopause was the most frequent menopausal-stage category (40.1%), followed by early perimenopause (21.1%), self-reported surgical menopause (19.8%), and late perimenopause (19.0%). Compared with the included participants, the 23 excluded participants had lower GSES total scores [median 27 (IQR 25–30.5) vs. 31 (IQR 28–36); *p* = 0.005] and a different distribution of menopausal stage (Fisher–Freeman–Halton exact *p* = 0.021), with early perimenopause being more frequent among excluded women (52.2% vs. 21.1%). No statistically significant between-group differences were detected for recruitment source or the remaining measured non-HADS variables (Supplementary Table S2).

Regarding psychological outcomes, 54.7% of participants had at least borderline anxiety symptoms (HADS-A ≥ 8), while 42.5% had at least borderline depressive symptoms (HADS-D ≥ 8). Scores at the lower boundary of 0 occurred in 4 participants (1.6%) for HADS-A and 14 participants (5.7%) for HADS-D, while the upper boundary of 21 occurred in 3 participants (1.2%) for HADS-A and none for HADS-D, indicating no substantial floor or ceiling concentration. The distributions of menopausal symptoms, menopause-related quality of life, self-efficacy, and psychological symptoms are presented in Table 2.

Spearman correlations of menopausal symptom burden, menopause-related quality of life, self-efficacy, and all 15 coping strategies with anxiety and depressive symptoms are presented in Table 3.

Psychological MRS scores showed the strongest correlations with anxiety (ρ = 0.550, q < 0.001) and depression (ρ = 0.492, q < 0.001). Nonpsychological MRS score, MENQOL total score, and GSES total score also remained associated with both outcomes after false-discovery-rate correction. Among the COPE subscales, venting of emotions showed the largest correlations with anxiety (ρ = 0.359, q < 0.001) and depression (ρ = 0.324, q < 0.001). Mental disengagement and behavioral disengagement were positively correlated with both outcomes after correction. Positive reinterpretation was inversely correlated with depression (ρ = −0.171, q = 0.019), but its nominal association with anxiety did not remain significant after correction (q = 0.109). The nominal association between denial and depression also did not remain significant after correction (q = 0.056). No other COPE subscale was associated with either outcome after false-discovery-rate correction. In the supplementary multivariable models containing all 15 COPE subscales simultaneously, no individual coping coefficient remained statistically significant after false-discovery-rate correction (all q ≥ 0.194). Nonpsychological MRS score remained associated with anxiety (B = 0.158, *p* = 0.003) and depression (B = 0.120, *p* = 0.027), while GSES score remained associated with depression (B = −0.201, *p* < 0.001) but not anxiety (B = −0.068, *p* = 0.222). Complete coping coefficients are presented in Supplementary Table S6. These analyses were treated as exploratory and were not used as the primary regression models.

Nested multivariable linear regression models were fitted separately for anxiety and depressive symptom severity. The symptom-burden models included nonpsychological MRS score and the measured sociodemographic and clinical covariates, while the psychological-resource models additionally included GSES score. Complete coefficients, standard errors, standardized coefficients, and model-fit statistics are presented in Table 4 and Supplementary Table S1.

For anxiety, the symptom-burden model explained 14.9% of the variance in HADS-A scores (adjusted R^2^ = 0.109). Greater nonpsychological MRS score was associated with greater anxiety severity (B = 0.243, 95% CI 0.142 to 0.345; β = 0.328; *p* < 0.001). After adding GSES score, nonpsychological MRS remained associated with anxiety (B = 0.231, 95% CI 0.130 to 0.332; β = 0.311; *p* < 0.001), and higher GSES score was associated with lower anxiety severity (B = −0.122, 95% CI −0.213 to −0.030; β = −0.169; *p* = 0.009). The psychological-resource model explained 17.5% of the variance (adjusted R^2^ = 0.133). Self-reported surgical menopause was associated with lower anxiety scores relative to early perimenopause (B = −2.396, 95% CI −4.218 to −0.573; *p* = 0.010), while the other measured covariates were not statistically significant.

For depression, the symptom-burden model explained 13.5% of the variance in HADS-D scores (adjusted R^2^ = 0.095). Greater nonpsychological MRS score was associated with greater depressive symptom severity (B = 0.202, 95% CI 0.093 to 0.311; β = 0.271; *p* < 0.001). In the psychological-resource model, nonpsychological MRS remained positively associated with depression (B = 0.179, 95% CI 0.077 to 0.281; β = 0.239; *p* < 0.001), while higher GSES score was inversely associated with depression (B = −0.231, 95% CI −0.313 to −0.150; β = −0.319; *p* < 0.001). This model explained 22.8% of the variance (adjusted R^2^ = 0.189). Self-reported surgical menopause was associated with lower depression scores relative to early perimenopause (B = −2.132, 95% CI −3.964 to −0.301; *p* = 0.023). The adjusted inverse association between GSES and HADS-D was significantly stronger than that between GSES and HADS-A (βHADS-D − βHADS-A = −0.150, bootstrap 95% CI −0.257 to −0.033, *p* = 0.008). There was no evidence that GSES modified the adjusted association between nonpsychological MRS score and either HADS-A (MRS × GSES interaction, *p* = 0.441) or HADS-D (*p* = 0.649). No other measured covariate was statistically significant.

Unstandardized coefficients are presented with HC3 robust standard errors in parentheses. The models included nonpsychological MRS score, GSES total score, age, BMI, menopausal stage, recruitment source, education, employment status, marital status, and comorbidity. Model fit was R^2^ = 0.175, adjusted R^2^ = 0.133, and RMSE = 3.977 for HADS-A and R^2^ = 0.228, adjusted R^2^ = 0.189, and RMSE = 3.868 for HADS-D. The maximum variance inflation factor was 2.510 in both models. Reference categories were early perimenopause, community recruitment, non-university education, not employed, single/divorced/widowed, and no comorbidity. Complete HC3 robust 95% confidence intervals and the symptom-burden models are reported in Supplementary Table S1.

The focal associations were also retained in the extended-covariate sensitivity models that additionally included residence, smoking status, menopause-related medication use, and SSRI or anxiolytic use. Nonpsychological MRS score remained associated with anxiety (B = 0.225, 95% CI 0.122 to 0.328; *p* < 0.001) and depression (B = 0.172, 95% CI 0.066 to 0.278; *p* = 0.002), while GSES score remained inversely associated with anxiety (B = −0.116, 95% CI −0.209 to −0.022; *p* = 0.016) and depression (B = −0.228, 95% CI −0.311 to −0.145; *p* < 0.001). Residence, smoking status, and the two medication indicators were not associated with either outcome (all *p* ≥ 0.159). Complete extended-model estimates are reported in Supplementary Table S1.

In the full analytical sample, the seven-item nonpsychological MRS composite showed acceptable internal consistency (Cronbach’s α = 0.773). Principal component analysis identified two components with eigenvalues greater than 1 (3.016 and 1.083), explaining 43.1% and 15.5% of the variance, respectively, and 58.6% cumulatively. The somatic and urogenital domain scores were moderately correlated (Spearman’s ρ = 0.543, *p* < 0.001). These findings indicate that the two domains are related but retain distinguishable symptom content, consistent with interpreting their sum as a multidomain index of nonpsychological symptom burden rather than as a strictly unidimensional scale.

In the full analytical sample, the somatic and urogenital MRS domains were each associated with anxiety and depressive symptom severity when entered separately (all *p* ≤ 0.006). When both domains were entered simultaneously, the urogenital domain remained associated with anxiety (B = 0.265, 95% CI 0.046 to 0.484; *p* = 0.018) and depression (B = 0.213, 95% CI 0.014 to 0.411; *p* = 0.036), whereas the somatic-domain coefficients were attenuated (*p* = 0.090 and *p* = 0.186, respectively). In the full analytical sample, exclusion of the sleep-problems item did not materially alter the association of the remaining six-item score with anxiety (B = 0.278, 95% CI 0.153 to 0.402; *p* < 0.001) or depression (B = 0.251, 95% CI 0.129 to 0.373; *p* < 0.001). In separate item-level models, sleep problems, joint and muscular discomfort, sexual problems, bladder problems, and vaginal dryness were associated with both outcomes after false-discovery-rate correction (all q ≤ 0.005), whereas hot flushes and heart discomfort were not. Supplementary Table S3 presents detailed domain- and item-level estimates; collectively, these analyses indicate that the composite association was not attributable solely to sleep problems. Participant characteristics according to menopausal-stage category are presented in Supplementary Table S4. In the restricted sensitivity analysis of women with natural postmenopause or self-reported surgical menopause and available data for years since menopause (n = 147), the surgical-menopause coefficient was attenuated and was no longer statistically significant for either anxiety (B = −1.310, 95% CI −3.339 to 0.719; *p* = 0.204) or depression (B = −1.149, 95% CI −3.021 to 0.723; *p* = 0.227). Years since menopause was not associated with anxiety (B = 0.078, 95% CI −0.132 to 0.287; *p* = 0.465) or depression (B = 0.023, 95% CI −0.181 to 0.226; *p* = 0.827). These results indicate that the original surgical-menopause coefficient was sensitive to the choice of reference group and adjustment for time since menopause.

Restricted cubic spline analyses provided no evidence of nonlinearity for nonpsychological MRS score, GSES score, or age in the anxiety model (*p* for nonlinearity = 0.658, 0.584, and 0.939, respectively) or the depression model (*p* = 0.492, 0.754, and 0.642, respectively). The proportional-odds models produced findings consistent with the linear regression analyses. For anxiety, each one-point increase in nonpsychological MRS score was associated with greater odds of being in a higher HADS-A category (OR = 1.132, 95% CI 1.079 to 1.189; *p* < 0.001), whereas each one-point increase in GSES score was associated with lower odds (OR = 0.955, 95% CI 0.915 to 0.997; *p* = 0.034). Corresponding associations were observed for depression for nonpsychological MRS score (OR = 1.096, 95% CI 1.043 to 1.152; *p* < 0.001) and GSES score (OR = 0.917, 95% CI 0.877 to 0.959; *p* < 0.001). Full model-specification sensitivity results are presented in Supplementary Table S5.

## 4. Discussion

This cross-sectional study of 247 peri- and postmenopausal women found that greater nonpsychological menopausal symptom burden and lower general self-efficacy were concurrently associated with greater anxiety and depressive symptom severity after adjustment for the measured sociodemographic and clinical characteristics. The standardized association of self-efficacy was stronger for depression than for anxiety, although the cross-sectional design does not establish temporal direction. Although self-reported surgical menopause was associated with lower psychological symptom scores relative to early perimenopause in the primary models, these coefficients were attenuated and no longer statistically significant when the analysis was restricted to natural and self-reported surgical postmenopause and adjusted for years since menopause. The exploratory coping analyses did not identify any individual COPE subscale that remained statistically significant in the simultaneous multivariable models after false-discovery-rate correction. Overall, the primary models explained a modest proportion of the variance in anxiety and depressive symptom severity.

Several interacting mechanisms may explain the association between nonpsychological menopausal symptoms and psychological distress. Fluctuations in ovarian hormones may affect thermoregulation, sleep, autonomic function, and responsiveness to psychosocial stress. In a longitudinal study, Gordon et al. reported that greater estradiol variability predicted subsequent depressive symptoms, particularly among women exposed to stressful life events, and was associated with heightened emotional sensitivity to social rejection [18]. These hormonal effects may contribute to psychological vulnerability both directly and indirectly through the symptoms experienced during the menopausal transition.

Menopausal symptoms might serve as an additional link connecting these biological changes to psychological effects. Vasomotor symptoms, musculoskeletal discomfort, fatigue, sleep issues, and urogenital problems can disrupt work, physical activity, focus, sexual health, and intimate relationships. Frequent nocturnal symptoms may lead to sleep fragmentation, impairing emotional regulation and increasing fatigue, irritability, and negative emotions. A recent study involving nearly 50,000 peri- and postmenopausal women found that sleep disturbances were linked to lower quality of life and higher depression and anxiety levels, regardless of the presence of vasomotor symptoms [19,20]. The concurrent association between the nonpsychological MRS score and HADS outcomes is consistent with symptom burden and psychological distress co-occurring, but does not establish the direction or mechanism of this relationship.

MENQOL total score was also moderately correlated with both HADS outcomes, indicating that greater overall menopause-related quality-of-life impairment co-occurred with greater psychological distress. Because MENQOL contains psychosocial, physical, vasomotor, and sexual content and may reflect both symptom burden and its consequences, this association was interpreted descriptively. Including MENQOL in the primary models could have adjusted for a construct partly overlapping with both the focal exposure and the outcomes; it was therefore retained as an exploratory measure rather than assigned a confounder, mediator, or competing-exposure role.

The current findings do not prove that menopausal symptoms directly cause anxiety or depression. Instead, psychological distress might heighten awareness of symptoms, reduce tolerance for physical discomfort, disrupt sleep, or increase the perceived impact of symptoms. Factors like hormonal fluctuations, sleep issues, psychosocial challenges, pre-existing mental health vulnerabilities, and how symptoms are perceived may all interact. Therefore, the observed relationship is likely bidirectional and involves multiple contributing factors.

Self-efficacy refers to an individual’s belief in their capability to organize and perform the actions required to manage a particular challenge. Within social cognitive theory, self-efficacy is proposed to influence goal setting, motivation, persistence, recovery from setbacks, and health-related behaviors [21]. Menopause-specific studies have reported associations between self-efficacy and psychological well-being, perceived stress, symptom management, symptom burden, self-compassion, and quality of life [22,23]. Structural equation and mediation analyses from previous research are consistent with self-efficacy functioning as a psychological resource; however, their directional interpretation depends on temporal and causal assumptions that cannot be tested using the present cross-sectional data. Psychological distress may reduce perceived capability, while self-efficacy and distress may also reflect shared underlying constructs such as perceived control, demoralization, or negative self-appraisal. Therefore, the current findings are interpreted as concurrent conditional associations rather than evidence that self-efficacy protects against psychological distress or mediates the effects of menopausal symptoms.

The present study extends previous research by examining anxiety and depressive symptoms separately while accounting for nonpsychological menopausal symptom burden and measured sociodemographic and clinical characteristics. In the psychological-resource models, higher GSES scores were concurrently associated with lower HADS-A and HADS-D scores. The standardized association was larger for depression than for anxiety, and a paired bootstrap comparison supported a difference between the two coefficients (Δβ for HADS-D versus HADS-A = −0.150, 95% CI −0.257 to −0.033; *p* = 0.008). This pattern may reflect the conceptual proximity of perceived self-efficacy to agency, motivation, persistence, and helplessness-related features of depression. However, it does not establish temporal direction: depressive symptoms may reduce confidence and perceived competence, and both constructs may be influenced by shared factors such as perceived control, demoralization, or negative self-appraisal. The findings therefore identify self-efficacy as a concurrent psychological correlate and do not demonstrate that it protects against depression or that targeting self-efficacy would improve psychological outcomes.

Several COPE subscales showed correlations with HADS outcomes, particularly mental disengagement, venting of emotions, and behavioral disengagement. However, in the exploratory multivariable models that included all 15 COPE subscales alongside nonpsychological MRS score, GSES score, and the measured sociodemographic and clinical covariates, no individual coping coefficient remained statistically significant after false-discovery-rate correction. These findings should therefore be interpreted as hypothesis-generating rather than as evidence for specific coping strategies.

This attenuation does not mean that coping is unimportant. Specific coping behaviors are situational and may vary according to the type, duration, and perceived controllability of a stressor.

The COPE inventory assesses the reported use of distinct coping responses but does not establish whether a response was appropriate for the particular stressor or whether it successfully reduced distress [16,24,25]. Coping effectiveness depends partly on flexibility and on the fit between the selected strategy and the demands or controllability of the situation; therefore, the same strategy may be adaptive in one context and ineffective in another [24,25]. Relationships between self-efficacy and coping may be bidirectional: perceived capability may shape the coping responses reported by participants, while coping experiences and psychological distress may also influence perceived capability. Because all constructs were measured concurrently, the present study cannot establish a hierarchy among self-efficacy, coping, and psychological adjustment. The observed pattern should therefore be interpreted as a difference in the consistency of concurrent associations, not as evidence that self-efficacy causally determines coping responses or psychological outcomes.

The lower anxiety and depression scores observed for self-reported surgical menopause relative to early perimenopause in the primary models should not be interpreted as evidence of a beneficial or protective association. The surgical-menopause category was based on participant self-report and could not be characterized according to exact procedure, surgical indication, confirmation of bilateral oophorectomy, or detailed hormone-therapy regimen. The group also differed in recruitment profile, with 77.6% recruited from the hospital or clinical setting, and may have differed in adaptation time and other unmeasured clinical characteristics. When the analysis was restricted to natural and self-reported surgical postmenopause and adjusted for years since menopause, the surgical-menopause coefficients were attenuated and were no longer statistically significant. The original finding may therefore reflect the choice of reference group, selection, adaptation, or residual confounding rather than a substantive effect of surgery.

More broadly, menopausal-stage categories are broad indicators of reproductive history and do not capture hormonal variability, individual hormone sensitivity, symptom interference, or psychosocial context. Women within the same category may therefore have markedly different symptom and psychological profiles. Brown et al. similarly reported that perceived control and symptom interference were associated with well-being, whereas menopausal stage was not [26]. Longitudinal studies using clinically verified surgical information are needed before psychological outcomes can be compared reliably across natural and surgical menopause.

Reported use of menopause-related medication and reported use of an SSRI or an anxiolytic were not independently associated with either HADS outcome in the extended sensitivity models. However, these exposures were recorded only as binary variables, without information on the specific agent or menopause-treatment class, therapeutic indication, dose, duration, adherence, or treatment response. The findings therefore indicate only that the principal associations were robust to coarse adjustment for reported medication use; they should not be interpreted as evidence of medication effectiveness or lack of effectiveness. Confounding by indication also remains likely because women with more severe symptoms may have been more likely to receive treatment.

Although these cross-sectional findings do not establish treatment effects, they highlight the clinical relevance of assessing menopausal symptom burden and psychological distress together. Clinical evaluation may include somatic and urogenital symptoms, sleep difficulties, anxiety, depressive symptoms, and perceived general self-efficacy. HADS remains a screening instrument rather than a diagnostic tool; women with elevated scores require appropriate clinical assessment rather than interpretation based solely on questionnaire results.

Intervention studies provide initial evidence that women’s perceptions and management of their symptoms can be modified clinically. Hunter summarized evidence indicating that menopause-specific cognitive behavioral therapy can reduce the impact of vasomotor symptoms and improve associated sleep and psychological outcomes [27]. A pilot randomized trial by Kim et al. showed improvements in menopausal symptoms, anxiety, somatic complaints, and overall quality of life after a menopause-adapted cognitive behavioral intervention [28]. Furthermore, Andrews et al. observed that structured symptom monitoring decreased physical symptoms and negative emotional reactions, even when accounting for coping styles [29]. While these studies do not establish that self-efficacy directly mediated these effects, they illustrate that altering symptom perception and management can impact both physical and emotional health.

The present findings generate several competing hypotheses for longitudinal research rather than establishing a directional mechanism. Hormonal and physiological changes may contribute to somatic, vasomotor, sleep-related, and urogenital symptoms that subsequently coincide with greater psychological distress. Conversely, anxiety or depressive symptoms may increase symptom perception and reporting, reduce perceived self-efficacy, or influence the coping responses used by participants. Menopausal symptoms, self-efficacy, coping, and psychological distress may also be jointly influenced by unmeasured clinical and psychosocial factors. Longitudinal studies with repeated measurements are therefore required to determine temporal ordering and to test whether changes in self-efficacy precede, follow, or occur concurrently with changes in psychological distress.

Several limitations should be noted. The cross-sectional design limits the ability to determine temporality, mediation, or causality. All key variables were self-reported, which could introduce recall, reporting, and common-method biases. Although removing the psychological MRS domain decreased item overlap, psychological state may still have affected reports of somatic and urogenital symptoms. Although the sensitivity analyses suggested that the associations were distributed across several somatic and urogenital symptoms and were not attributable solely to sleep problems, the item-level findings remain exploratory. General self-efficacy was assessed, not efficacy specific to menopause or symptom management. Similarly, coping measures did not evaluate the context, suitability, or effectiveness of strategies. Residual confounding remains possible because psychiatric history, previous depressive or anxiety episodes, diagnosed sleep disorders, stressful life events, social support, financial strain, relationship dynamics, and hormone levels were not collected. Current SSRI or anxiolytic use was available but cannot be considered a reliable substitute for psychiatric history because treatment indication, timing, duration, and adherence were unknown. Education, employment, marital status, residence, smoking, comorbidity, and medication indicators were examined in the primary or extended-covariate models, but adjustment for these measured characteristics cannot eliminate confounding by the unmeasured clinical and psychosocial factors. The exploratory coping analyses were adjusted for multiple testing using the Benjamini–Hochberg false-discovery-rate procedure; nevertheless, they remain hypothesis-generating because they were not formally preregistered. Medication data lacked detail for treatment comparison, and the models explained only a modest part of the variation in anxiety and depression. The exclusion of 23 participants whose HADS scores could not be independently verified may also have introduced selection bias. Although most observed characteristics did not differ significantly between the included and excluded groups, the excluded participants had a different menopausal-stage distribution and lower general self-efficacy scores. Therefore, associations involving GSES may be particularly sensitive to this exclusion, and the direction or magnitude of any resulting bias cannot be determined because the HADS outcomes of the excluded participants could not be independently verified. Lastly, the sample was predominantly urban, employed, university-educated, and married or partnered. This socioeconomically advantaged profile may have contributed to the relatively high GSES scores by overrepresenting women with greater access to informational, social, and material resources and may also have restricted the variability of self-efficacy within the sample. Although recruitment from both hospital and community settings increased sample diversity, the nonprobability recruitment approach limits generalizability, particularly to Romanian women living in rural areas, those with lower educational or socioeconomic status, and those receiving specialist psychiatric care.

## 5. Conclusions

Greater nonpsychological menopausal symptom burden and lower general self-efficacy were concurrently associated with greater anxiety and depressive symptom severity. The association with self-efficacy was stronger for depression, although temporal direction cannot be determined. The surgical-menopause finding was attenuated in sensitivity analysis and should not be interpreted as protective; longitudinal confirmation is required.

## Figures and Tables

**Table 1:** Participant characteristics (*N* = 247).

Characteristic	Value
Age, years	51 (48–55)
BMI, kg/m^2^	25.3 (22.4–28.3)
Urban residence	178 (72.1%)
University education	159 (64.4%)
Employed	204 (82.6%)
Married/partnered	196 (79.4%)
Recruitment source, community/hospital-based	159 (64.4%)/88 (35.6%)
Menopausal stage, early/late/natural postmenopause/self-reported surgical menopause	52 (21.1%)/47 (19.0%)/99 (40.1%)/49 (19.8%)
Smoking, never/former/current	118 (47.8%)/65 (26.3%)/64 (25.9%)
Any comorbidity	108 (43.7%)
Menopause-related medication	56 (22.7%)
SSRI or anxiolytic use	27 (10.9%)

Data are presented as median (interquartile range) or n (%). Abbreviations: BMI, body mass index; IQR, interquartile range; SSRI, selective serotonin reuptake inhibitor.

**Table 2:** Distribution of symptom burden, quality of life, psychological outcomes, and self-efficacy (*N* = 247).

Measure	Value
MRS somatic	6 (4–8)
MRS psychological	5 (3–9)
MRS urogenital	5 (2–7)
MRS total	17 (9–22.8)
MRS nonpsychological	11 (6–15)
MENQOL vasomotor	2.67 (1.00–5.00)
MENQOL psychosocial	2.86 (1.71–4.57)
MENQOL physical	3.88 (2.63–4.75)
MENQOL sexual	4.00 (1.00–6.00)
MENQOL total	3.59 (2.52–4.63)
GSES total	31 (28–36)
HADS anxiety	8 (5–11)
HADS depression	6 (3–10)
HADS anxiety category, normal/borderline/clinical	112 (45.3%)/70 (28.3%)/65 (26.3%)
HADS depression category, normal/borderline/clinical	142 (57.5%)/55 (22.3%)/50 (20.2%)

Data are presented as median (interquartile range) or n (%). The nonpsychological MRS score was calculated as the sum of the somatic and urogenital domains. HADS categories were defined as normal (0–7), borderline (8–10), and clinical range (11–21). Abbreviations: GSES, General Self-Efficacy Scale; HADS, Hospital Anxiety and Depression Scale; IQR, interquartile range; MENQOL, Menopause-Specific Quality of Life Questionnaire; MRS, Menopause Rating Scale.

**Table 3:** Spearman correlations of symptom burden, quality of life, self-efficacy, and coping strategies with anxiety and depressive symptoms (*N* = 247).

Variable	HADS-A, ρ (95% CI)	*p*-Value	q-Value	HADS-D, ρ (95% CI)	*p*-Value	q-Value
MRS psychological	0.550 (0.456 to 0.631)	<0.001	<0.001	0.492 (0.392 to 0.581)	<0.001	<0.001
MRS nonpsychological	0.357 (0.242 to 0.461)	<0.001	<0.001	0.287 (0.168 to 0.397)	<0.001	<0.001
MENQOL total	0.396 (0.285 to 0.496)	<0.001	<0.001	0.347 (0.233 to 0.453)	<0.001	<0.001
GSES total	−0.186 (−0.304 to −0.063)	0.003	0.011	−0.373 (−0.476 to −0.261)	<0.001	<0.001
COPE positive reinterpretation	−0.127 (−0.248 to −0.002)	0.046	0.109	−0.171 (−0.290 to −0.047)	0.007	0.019
COPE mental disengagement	0.216 (0.094 to 0.332)	<0.001	0.002	0.214 (0.092 to 0.330)	<0.001	0.002
COPE venting of emotions	0.359 (0.245 to 0.463)	<0.001	<0.001	0.324 (0.208 to 0.431)	<0.001	<0.001
COPE instrumental support	0.000 (−0.125 to 0.125)	0.998	0.998	0.024 (−0.102 to 0.148)	0.713	0.820
COPE active coping	0.101 (−0.024 to 0.223)	0.113	0.196	0.015 (−0.111 to 0.139)	0.820	0.820
COPE denial	0.102 (−0.023 to 0.224)	0.110	0.196	0.141 (0.016 to 0.261)	0.027	0.056
COPE religion	0.078 (−0.047 to 0.201)	0.223	0.326	0.052 (−0.073 to 0.176)	0.413	0.524
COPE humor	−0.085 (−0.208 to 0.040)	0.181	0.286	−0.113 (−0.235 to 0.012)	0.075	0.130
COPE behavioral disengagement	0.182 (0.059 to 0.300)	0.004	0.011	0.158 (0.034 to 0.278)	0.013	0.030
COPE restraint	0.103 (−0.022 to 0.225)	0.107	0.196	0.114 (−0.011 to 0.235)	0.074	0.130
COPE emotional support	−0.027 (−0.152 to 0.098)	0.670	0.848	0.017 (−0.108 to 0.142)	0.787	0.820
COPE substance use	0.007 (−0.118 to 0.132)	0.914	0.998	0.078 (−0.047 to 0.201)	0.222	0.352
COPE acceptance	−0.001 (−0.126 to 0.124)	0.991	0.998	−0.062 (−0.185 to 0.064)	0.335	0.489
COPE suppression of competing activities	0.057 (−0.068 to 0.181)	0.371	0.504	0.016 (−0.109 to 0.141)	0.800	0.820
COPE planning	0.004 (−0.121 to 0.129)	0.946	0.998	−0.057 (−0.181 to 0.068)	0.372	0.505

Values are Spearman rank correlation coefficients with 95% confidence intervals. Nominal *p*-values and Benjamini–Hochberg false-discovery-rate-adjusted q-values are reported. The false-discovery-rate correction was applied separately for HADS-A and HADS-D across the complete family of 19 correlation tests. All tests were two-sided. Abbreviations: CI, confidence interval; COPE, Coping Orientation to Problems Experienced; GSES, General Self-Efficacy Scale; HADS-A, Hospital Anxiety and Depression Scale–Anxiety; HADS-D, Hospital Anxiety and Depression Scale–Depression; MENQOL, Menopause-Specific Quality of Life Questionnaire; MRS, Menopause Rating Scale; ρ, Spearman rank correlation coefficient.

**Table 4:** Psychological-resource regression models for anxiety and depressive symptom severity (*N* = 247).

Predictor	HADS-A, B3.	β	*p*-Value	HADS-D, B (HC3 SE)	β	*p*-Value
MRS nonpsychological score	0.231 (0.051)	0.311	<0.001	0.179 (0.052)	0.239	<0.001
GSES total score	−0.122 (0.047)	−0.169	0.009	−0.231 (0.041)	−0.319	<0.001
Age, years	0.016 (0.064)	0.018	0.807	−0.033 (0.056)	−0.039	0.550
BMI, kg/m^2^	0.109 (0.076)	0.111	0.150	0.100 (0.060)	0.100	0.099
Late perimenopause	−0.875 (0.874)	−0.081	0.317	−1.250 (0.864)	−0.115	0.149
Natural postmenopause	−1.290 (0.926)	−0.148	0.165	−1.089 (0.874)	−0.125	0.214
Self-reported surgical menopause	−2.396 (0.925)	−0.224	0.010	−2.132 (0.930)	−0.198	0.023
Hospital/clinical recruitment	0.400 (0.646)	0.045	0.536	0.021 (0.610)	0.002	0.973
University education	0.244 (0.558)	0.027	0.663	−0.233 (0.553)	−0.026	0.674
Employed	−0.349 (0.670)	−0.031	0.604	−0.898 (0.610)	−0.079	0.143
Married/partnered	−0.494 (0.675)	−0.047	0.465	−0.382 (0.696)	−0.036	0.583
Any comorbidity	−0.398 (0.544)	−0.046	0.465	0.087 (0.547)	0.010	0.874

Abbreviations: B, unstandardized regression coefficient; BMI, body mass index; GSES, General Self-Efficacy Scale; HADS-A, Hospital Anxiety and Depression Scale–Anxiety; HADS-D, Hospital Anxiety and Depression Scale–Depression; MRS, Menopause Rating Scale; RMSE, root mean square error; SE, standard error; β, standardized regression coefficient.

## Data Availability

The data are not publicly available because they contain participant-level health information, and public sharing was not covered by the informed consent procedure. Requests for access may be directed to the corresponding author and will be considered in accordance with participant consent, institutional policies, and applicable data-protection requirements.

## Supplementary Materials

The following supporting information can be downloaded at: https://www.mdpi.com/article/10.3390/jcm15186944/s1, Figure S1: Directed acyclic graph illustrating the conceptual relationships used to organize the multivariable analyses; Table S1: Complete multivariable linear regression models for anxiety and depressive symptom severity; Table S2: Comparison of participants included in and excluded from the outcome analyses; Table S3: Sensitivity analyses of the composition of the nonpsychological MRS score; Table S4: Characteristics of participants according to menopausal-stage category; Table S5: Sensitivity analyses of functional form and HADS outcome specification; Table S6: Exploratory multivariable associations of all 15 COPE subscales with anxiety and depressive symptom severity.

## Abbreviations

The following abbreviations are used in this manuscript:

BMI	Body mass index
CI	Confidence interval
COPE	Coping Orientation to Problems Experienced Inventory
GSES	General Self-Efficacy Scale
HADS	Hospital Anxiety and Depression Scale
HADS-A	Hospital Anxiety and Depression Scale–Anxiety subscale
HADS-D	Hospital Anxiety and Depression Scale–Depression subscale
HC3	Heteroscedasticity-consistent type 3
IQR	Interquartile range
MENQOL	Menopause-Specific Quality of Life Questionnaire
MRS	Menopause Rating Scale
Q–Q	Quantile–quantile
SSRI	Selective serotonin reuptake inhibitor
STRAW+10	Stages of Reproductive Aging Workshop +10
STROBE	Strengthening the Reporting of Observational Studies in Epidemiology
SWAN	Study of Women’s Health Across the Nation
