# Supplementary material for: Beyond Menopausal Stage: Symptom Burden, Self-Efficacy, and Psychological Distress
Source: J Clin Med. 2026 Sep 8;15(18):6944. doi: 10.3390/jcm15186944 (PMC13607617; doi:10.3390/jcm15186944)
Supplement: Supplementary file 1 [file jcm-15-06944-s001.zip › jcm-4533059-supplementary.pdf]

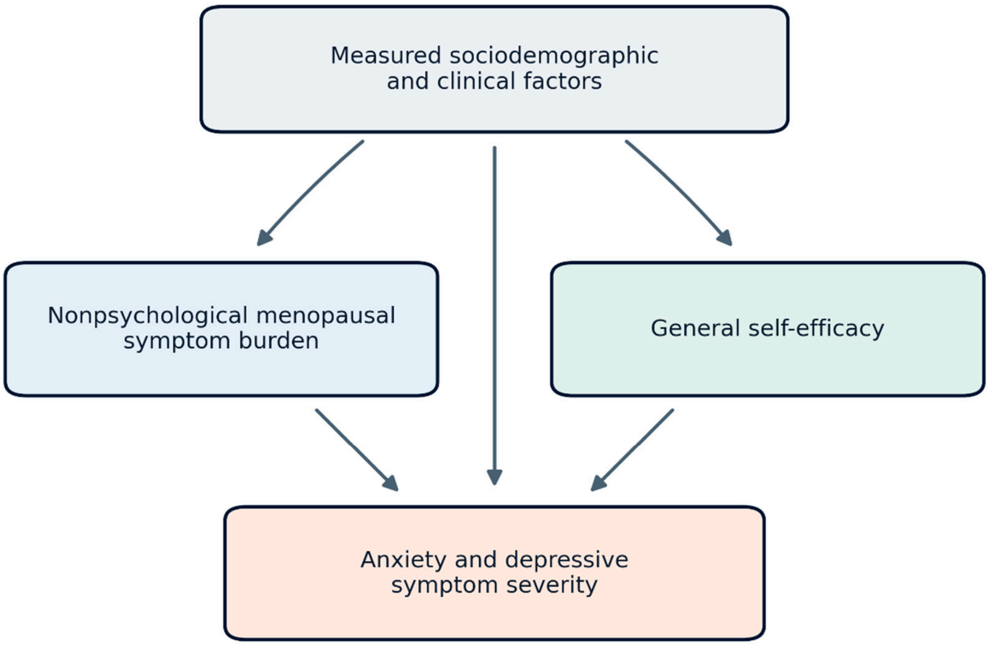

Supplementary Figure S1. Directed acyclic graph illustrating the conceptual relationships used to organize the multivariable analyses.
Arrows represent hypothesized relationships used to organize covariate selection and do not indicate causal effects established by the present cross-sectional data. Sociodemographic and clinical factors included age, body mass index, menopausal stage, recruitment source, education, employment status, marital status, and comorbidity. Reverse directionality between psychological distress, symptom reporting, and perceived self-efficacy remains possible.

Supplementary Table S1. Complete multivariable linear regression models for anxiety and depressive symptom severity (N = 247)

**Predictor**	**HADS-A, B (95% CI)**	**β**	**p-value**	**HADS-D, B (95% CI)**	**β**	**p-value**
**Model 1. Symptom-burden model**						
MRS nonpsychological score	0.243 (0.142 to 0.345)	0.328	<0.001	0.202 (0.093 to 0.311)	0.271	<0.001
Age, years	0.017 (−0.111 to 0.145)	0.020	0.794	−0.031 (−0.156 to 0.094)	−0.036	0.628
BMI, kg/m²	0.093 (−0.058 to 0.243)	0.094	0.227	0.069 (−0.060 to 0.197)	0.069	0.295
Late perimenopause	−0.957 (−2.657 to 0.743)	−0.088	0.268	−1.405 (−3.179 to 0.369)	−0.129	0.120

Natural postmenopause	−1.500 (−3.275 to 0.275)	−0.172	0.097	−1.488 (−3.226 to 0.249)	−0.170	0.093
Self-reported surgical menopause	−2.565 (−4.372 to −0.759)	−0.240	0.006	−2.454 (−4.395 to −0.514)	−0.228	0.013
Hospital/clinical recruitment	0.173 (−1.111 to 1.458)	0.019	0.790	−0.410 (−1.673 to 0.853)	−0.046	0.523
University education	0.043 (−1.052 to 1.138)	0.005	0.938	−0.614 (−1.739 to 0.510)	−0.069	0.283
Employed	−0.200 (−1.559 to 1.159)	−0.018	0.772	−0.616 (−1.974 to 0.743)	−0.054	0.373
Married/partnered	−0.394 (−1.705 to 0.916)	−0.037	0.554	−0.193 (−1.619 to 1.233)	−0.018	0.790
Any comorbidity	−0.346 (−1.419 to 0.728)	−0.040	0.527	0.186 (−0.948 to 1.320)	0.022	0.747
**Model fit:** HADS-A, R^2 = 0.149, adjusted R^2 = 0.109, RMSE = 4.031; HADS-D, R^2 = 0.135, adjusted R^2 = 0.095, RMSE = 4.085. Maximum VIF = 2.487.						
**Model 2. Psychological-resource model**						
MRS nonpsychological score	0.231 (0.130 to 0.332)	0.311	<0.001	0.179 (0.077 to 0.281)	0.239	<0.001
GSES total score	−0.122 (−0.213 to −0.030)	−0.169	0.009	−0.231 (−0.313 to −0.150)	−0.319	<0.001
Age, years	0.016 (−0.110 to 0.141)	0.018	0.807	−0.033 (−0.143 to 0.076)	−0.039	0.550
BMI, kg/m²	0.109 (−0.040 to 0.258)	0.111	0.150	0.100 (−0.019 to 0.218)	0.100	0.099
Late perimenopause	−0.875 (−2.597 to 0.845)	−0.081	0.317	−1.250 (−2.953 to 0.452)	−0.115	0.149
Natural postmenopause	−1.290 (−3.114 to 0.534)	−0.148	0.165	−1.089 (−2.811 to 0.633)	−0.125	0.214
Self-reported surgical menopause	−2.396 (−4.218 to −0.573)	−0.224	0.010	−2.132 (−3.964 to −0.301)	−0.198	0.023
Hospital/clinical recruitment	0.400 (−0.871 to 1.672)	0.045	0.536	0.021 (−1.180 to 1.222)	0.002	0.973
University education	0.244 (−0.856 to 1.344)	0.027	0.663	−0.233 (−1.323 to 0.857)	−0.026	0.674
Employed	−0.349 (−1.669 to 0.972)	−0.031	0.604	−0.898 (−2.100 to 0.304)	−0.079	0.143
Married/partnered	−0.494 (−1.824 to 0.836)	−0.047	0.465	−0.382 (−1.753 to 0.988)	−0.036	0.583
Any comorbidity	−0.398 (−1.469 to 0.673)	−0.046	0.465	0.087 (−0.991 to 1.164)	0.010	0.874

**Model fit:** HADS-A, R^2 = 0.175, adjusted R^2 = 0.133, RMSE = 3.977; HADS-D, R^2 = 0.228, adjusted R^2 = 0.189, RMSE = 3.868. Maximum VIF = 2.510.						
**Model 3. Extended-covariate sensitivity model**						
MRS nonpsychological score	0.225 (0.122 to 0.328)	0.303	<0.001	0.172 (0.066 to 0.278)	0.230	0.002
GSES total score	−0.116 (−0.209 to −0.022)	−0.160	0.016	−0.228 (−0.311 to −0.145)	−0.315	<0.001
Age, years	0.026 (−0.104 to 0.155)	0.030	0.696	−0.023 (−0.137 to 0.090)	−0.027	0.686
BMI, kg/m^2	0.135 (−0.022 to 0.292)	0.137	0.092	0.129 (0.005 to 0.253)	0.130	0.042
Late perimenopause	−1.035 (−2.823 to 0.753)	−0.095	0.255	−1.506 (−3.250 to 0.238)	−0.138	0.090
Natural postmenopause	−1.315 (−3.208 to 0.579)	−0.151	0.173	−1.099 (−2.840 to 0.642)	−0.126	0.215
Self-reported surgical menopause	−2.478 (−4.354 to −0.602)	−0.232	0.010	−2.141 (−4.012 to −0.271)	−0.199	0.025
Hospital/clinical recruitment	0.568 (−0.730 to 1.867)	0.064	0.389	0.225 (−1.017 to 1.466)	0.025	0.722
University education	0.208 (−0.965 to 1.381)	0.023	0.727	−0.174 (−1.352 to 1.004)	−0.019	0.771
Employed	−0.334 (−1.705 to 1.037)	−0.030	0.632	−0.868 (−2.072 to 0.336)	−0.077	0.157
Married/partnered	−0.486 (−1.823 to 0.852)	−0.046	0.475	−0.381 (−1.748 to 0.986)	−0.036	0.583
Any comorbidity	−0.340 (−1.456 to 0.776)	−0.040	0.549	0.160 (−0.935 to 1.255)	0.019	0.774
Urban residence	0.629 (−0.583 to 1.841)	0.066	0.307	0.525 (−0.592 to 1.642)	0.055	0.355
Former smoker	0.026 (−1.284 to 1.336)	0.003	0.969	0.579 (−0.658 to 1.817)	0.060	0.357
Current smoker	0.324 (−1.043 to 1.691)	0.033	0.641	0.931 (−0.368 to 2.229)	0.095	0.159
Menopause-related medication	0.627 (−0.773 to 2.027)	0.062	0.378	0.648 (−0.750 to 2.047)	0.063	0.362
SSRI or anxiolytic use	0.584 (−1.110 to 2.279)	0.043	0.497	0.746 (−0.882 to 2.373)	0.054	0.368
**Model fit:** HADS-A, R^2 = 0.185, adjusted R^2 = 0.124, RMSE = 3.997; HADS-D, R^2 = 0.246, adjusted R^2 = 0.190, RMSE = 3.865. Maximum VIF = 2.624.<br>Abbreviations: B, unstandardized regression coefficient; BMI, body mass index; CI, confidence interval; GSES, General Self-Efficacy Scale; HADS-A, Hospital Anxiety and Depression Scale–Anxiety; HADS-D,						

Hospital Anxiety and Depression Scale–Depression; HC3, heteroscedasticity-consistent covariance matrix estimator 3; MRS, Menopause Rating Scale; RMSE, root mean square error; SSRI, selective serotonin reuptake inhibitor; VIF, variance inflation factor; β, standardized regression coefficient.

Values are unstandardized regression coefficients (B) with HC3 heteroscedasticity-consistent 95% confidence intervals, standardized regression coefficients (β), and two-sided p-values. All predictors within each model were entered simultaneously. The symptom-burden model included the nonpsychological MRS score and the measured sociodemographic and clinical covariates. The psychological-resource model additionally included GSES total score. The extended-covariate sensitivity model additionally included residence, smoking status, menopause-related medication use, and SSRI or anxiolytic use. Menopausal-stage categories were mutually exclusive; participants reporting surgical menopause were assigned to the self-reported surgical-menopause category. One missing BMI value was replaced with the sample median for the multivariable analyses. Reference categories were early perimenopause, community recruitment, non-university education, not employed, single/divorced/widowed, and no comorbidity. The extended-covariate sensitivity model additionally used rural residence, never smoking, no menopause-related medication, and no SSRI or anxiolytic use as reference categories.

Supplementary Table S2. Comparison of participants included in and excluded from the outcome analyses

**Characteristic**	**Included (n = 247)**	**Excluded (n = 23)**	**p-value**
Age, years	51 (48–55)	49 (48–51.5)	0.056
BMI, kg/m²	25.3 (22.4–28.3)	25.0 (22.6–26.3)	0.420
Recruitment source			0.821
Community	159 (64.4%)	14 (60.9%)	
Hospital/clinical setting	88 (35.6%)	9 (39.1%)	
Urban residence	178 (72.1%)	16 (69.6%)	0.810
Education			0.310
University	159 (64.4%)	12 (52.2%)	
High school	79 (32.0%)	11 (47.8%)	
Middle school	9 (3.6%)	0 (0.0%)	
Employed	204 (82.6%)	20 (87.0%)	0.776
Married/partnered	196 (79.4%)	19 (82.6%)	1.000
Menopausal stage			0.021
Early perimenopause	52 (21.1%)	12 (52.2%)	
Late perimenopause	47 (19.0%)	2 (8.7%)	
Natural postmenopause	99 (40.1%)	6 (26.1%)	
Surgical menopause	49 (19.8%)	3 (13.0%)	
Smoking status			0.717
Never	118 (47.8%)	13 (56.5%)	
Former	65 (26.3%)	4 (17.4%)	

Current	64 (25.9%)	6 (26.1%)	
Any comorbidity	108 (43.7%)	13 (56.5%)	0.276
Menopause-related medication	56 (22.7%)	7 (30.4%)	0.440
SSRI or anxiolytic use	27 (10.9%)	1 (4.3%)	0.486
MRS nonpsychological score	11 (6–15)	13 (10–17)	0.202
MENQOL total score	3.59 (2.53–4.63)	3.99 (2.30–4.54)	0.955
GSES total score	31 (28–36)	27 (25–30.5)	0.005
Abbreviations: BMI, body mass index; GSES, General Self-Efficacy Scale; IQR, interquartile range; MENQOL, Menopause-Specific Quality of Life Questionnaire; MRS, Menopause Rating Scale; SSRI, selective serotonin reuptake inhibitor.			

Data are presented as median (interquartile range) or n (%). Continuous variables were compared using the Mann–Whitney U test. Categorical variables were compared using Fisher's exact test or the Fisher–Freeman–Halton exact test, as appropriate. BMI was available for 246 included participants and all 23 excluded participants; the single missing BMI value was imputed only for the multivariable regression analyses and not for this comparison. Abbreviations: BMI, body mass index; GSES, General Self-Efficacy Scale; MENQOL, Menopause-Specific Quality of Life Questionnaire; MRS, Menopause Rating Scale; SSRI, selective serotonin reuptake inhibitor.

Supplementary Table S3. Sensitivity analyses of the composition of the nonpsychological MRS score
Panel A. Domain-level sensitivity analyses in the examined sample (N = 247)

**Model and MRS component**	**HADS-A, B (95% CI)**	**p-value**	**HADS-D, B (95% CI)**	**p-value**
Somatic domain entered separately	0.341 (0.154 to 0.527)	<0.001	0.260 (0.074 to 0.445)	0.006
Urogenital domain entered separately	0.366 (0.187 to 0.546)	<0.001	0.287 (0.115 to 0.459)	0.001
Somatic domain, both domains entered	0.196 (−0.031 to 0.423)	0.090	0.144 (−0.070 to 0.357)	0.186
Urogenital domain, both domains entered	0.265 (0.046 to 0.484)	0.018	0.213 (0.014 to 0.411)	0.036

Panel B. Item-level sensitivity analyses in participants' MRS item responses (N = 247)

**MRS score or item**	**HADS-A, B (95% CI)**	**p-value**	**q-value**	**HADS-D, B (95% CI)**	**p-value**	**q-value**
Six-item score excluding sleep problems	0.278 (0.153 to 0.402)	<0.001	—	0.251 (0.129 to 0.373)	<0.001	—
Hot flushes and sweating	0.238 (−0.256 to 0.731)	0.343	0.343	0.035 (−0.426 to 0.496)	0.882	0.882
Heart discomfort	0.402 (−0.144 to 0.947)	0.148	0.173	0.265 (−0.318 to 0.847)	0.371	0.433

Sleep problems	0.928 (0.457 to 1.399)	<0.001	<0.001	0.679 (0.243 to 1.114)	0.002	0.004
Joint and muscular discomfort	0.820 (0.273 to 1.367)	0.003	0.005	0.874 (0.369 to 1.378)	0.001	0.002
Sexual problems	0.945 (0.467 to 1.423)	<0.001	<0.001	0.927 (0.478 to 1.375)	<0.001	<0.001
Bladder problems	0.821 (0.311 to 1.332)	0.002	0.003	0.808 (0.365 to 1.251)	<0.001	0.001
Vaginal dryness	0.723 (0.308 to 1.137)	0.001	0.002	0.623 (0.209 to 1.037)	0.003	0.005

Abbreviations: B, unstandardized regression coefficient; CI, confidence interval; HADS-A, Hospital Anxiety and Depression Scale–Anxiety; HADS-D, Hospital Anxiety and Depression Scale–Depression; MRS, Menopause Rating Scale.

Values are unstandardized regression coefficients with HC3 robust 95% confidence intervals. All models were adjusted for general self-efficacy, age, body mass index, menopausal stage, recruitment source, education, employment status, marital status, and comorbidity. In Panel A, the somatic and urogenital domains were first examined in separate models and were subsequently entered simultaneously. In Panel B, each individual MRS item was examined in a separate adjusted model. Benjamini–Hochberg false-discovery-rate correction was applied across the seven item-level tests separately for HADS-A and HADS-D; q-values were not calculated for the six-item sensitivity score. Physical and mental exhaustion was not included because it belongs to the psychological MRS domain.

Supplementary Table S4. Characteristics of participants according to menopausal-stage category

**Characteristic**	**Early perimenopause (n = 52)**	**Late perimenopause (n = 47)**	**Natural postmenopause (n = 99)**	**Self-reported surgical menopause (n = 49)**
Age, years	49 (46–50)	50 (47.5–54)	55 (52–58)	50 (47–54)
BMI, kg/m²	24.8 (22.0–26.9)	25.2 (23.4–28.4)	25.3 (22.4–27.2)	25.9 (23.5–30.8)
Years since menopause	—	—	5 (2–10)	2 (0–4)
Hospital/clinical recruitment	12 (23.1%)	13 (27.7%)	25 (25.3%)	38 (77.6%)
Any comorbidity	26 (50.0%)	21 (44.7%)	34 (34.3%)	27 (55.1%)
Menopause-related medication	15 (28.8%)	17 (36.2%)	14 (14.1%)	10 (20.4%)
SSRI or anxiolytic use	3 (5.8%)	8 (17.0%)	12 (12.1%)	4 (8.2%)
MRS nonpsychological score	8.5 (4–14)	13 (7.5–16)	12 (6.5–16)	9 (5–13)

GSES total score	30 (27–34)	31 (28–37)	32 (29–36.5)	34 (30–37)
HADS-A score	9 (6–11)	9 (6.5–11)	8 (5–11)	6 (4–10)
HADS-D score	7.5 (5–11)	7 (4–10)	6 (4–10)	5 (2–9)
Abbreviations: BMI, body mass index; GSES, General Self-Efficacy Scale; HADS-A, Hospital Anxiety and Depression Scale–Anxiety; HADS-D, Hospital Anxiety and Depression Scale–Depression; IQR, interquartile range; MRS, Menopause Rating Scale; SSRI, selective serotonin reuptake inhibitor.				

Data are presented as median (interquartile range) or n (%). Years since menopause was summarized only for the natural-postmenopause and self-reported surgical-menopause categories and was available for 99 and 48 participants, respectively. BMI was unavailable for one participant with natural postmenopause. The questionnaire did not collect the indication for surgery, the exact surgical procedure, confirmation of bilateral oophorectomy, or detailed hormone-therapy regimens. The surgical-menopause category was therefore based on participant self-report.

Supplementary Table S5. Sensitivity analyses of functional form and HADS outcome specification

Panel A. Restricted cubic spline tests of nonlinearity

**Predictor**	**Knot locations**	**HADS-A, p for nonlinearity**	**HADS-D, p for nonlinearity**
MRS nonpsychological score	2, 8, 13, 20	0.658	0.492
GSES total score	20, 30, 34, 40	0.584	0.754
Age, years	45, 50, 54, 59.7	0.939	0.642

Panel B. Proportional-odds ordinal logistic regression models

**Predictor**	**HADS-A, OR (95% CI)**	**p-value**	**HADS-D, OR (95% CI)**	**p-value**
MRS nonpsychological score	1.132 (1.079 to 1.189)	<0.001	1.096 (1.043 to 1.152)	<0.001
GSES total score	0.955 (0.915 to 0.997)	0.034	0.917 (0.877 to 0.959)	<0.001
Age, years	1.017 (0.957 to 1.081)	0.580	0.978 (0.919 to 1.041)	0.492
BMI, kg/m^2	1.061 (0.998 to 1.128)	0.060	1.035 (0.972 to 1.102)	0.283
Late perimenopause	0.635 (0.285 to 1.415)	0.266	0.708 (0.313 to 1.599)	0.406
Natural postmenopause	0.449 (0.202 to 0.996)	0.049	0.596 (0.264 to 1.348)	0.214
Self-reported surgical menopause	0.323 (0.137 to 0.761)	0.010	0.499 (0.203 to 1.226)	0.130
Hospital/clinical recruitment	1.101 (0.619 to 1.959)	0.743	1.032 (0.567 to 1.879)	0.918

University education	1.217 (0.725 to 2.043)	0.458	0.933 (0.543 to 1.604)	0.803
Employed	0.648 (0.330 to 1.272)	0.207	0.741 (0.369 to 1.489)	0.400
Married/partnered	0.782 (0.417 to 1.464)	0.442	1.018 (0.514 to 2.015)	0.959
Any comorbidity	0.863 (0.519 to 1.435)	0.570	0.952 (0.558 to 1.624)	0.856
Abbreviations: BMI, body mass index; CI, confidence interval; GSES, General Self-Efficacy Scale; HADS-A, Hospital Anxiety and Depression Scale–Anxiety; HADS-D, Hospital Anxiety and Depression Scale–Depression; MRS, Menopause Rating Scale; OR, odds ratio.				

In Panel A, four-knot restricted cubic splines were fitted at the 5th, 35th, 65th, and 95th percentiles of each predictor. Reported p-values are from joint Wald tests of the nonlinear spline terms. In Panel B, odds ratios greater than 1 indicate greater odds of being in a higher HADS symptom category. Odds ratios for continuous predictors represent a one-unit increase. HADS categories were defined as normal (0–7), borderline (8–10), and clinical range (11–21). All models included the predictors listed in Panel B. Reference categories were early perimenopause, community recruitment, non-university education, not employed, single/divorced/widowed, and no comorbidity.

Supplementary Table S6. Exploratory multivariable associations of all 15 COPE subscales with anxiety and depressive symptom severity

**COPE subscale**	**HADS-A, B (95% CI)**	**p-value**	**q-value**	**HADS-D, B (95% CI)**	**p-value**	**q-value**
Positive reinterpretation	−1.545 (−3.009 to −0.081)	0.039	0.194	−1.559 (−3.021 to −0.098)	0.037	0.549
Mental disengagement	0.548 (−0.657 to 1.753)	0.371	0.701	0.784 (−0.397 to 1.966)	0.192	0.770
Venting of emotions	1.195 (0.188 to 2.201)	0.020	0.194	0.545 (−0.409 to 1.499)	0.261	0.770
Instrumental support	−0.059 (−1.146 to 1.027)	0.914	0.914	0.224 (−0.955 to 1.404)	0.708	0.817
Active coping	1.679 (0.138 to 3.221)	0.033	0.194	1.216 (−0.293 to 2.726)	0.114	0.770
Denial	−0.309 (−1.357 to 0.740)	0.562	0.703	0.438 (−0.750 to 1.627)	0.468	0.817
Religion	0.277 (−0.327 to 0.881)	0.367	0.701	0.196 (−0.412 to 0.804)	0.525	0.817
Humor	−0.417 (−1.178 to 0.345)	0.282	0.701	−0.362 (−1.077 to 0.352)	0.319	0.770
Behavioral disengagement	0.496 (−0.767 to 1.759)	0.440	0.701	−0.294 (−1.536 to 0.949)	0.642	0.817

Restraint	0.418 (−0.840 to 1.675)	0.514	0.701	0.771 (−0.483 to 2.024)	0.227	0.770
Emotional support	−0.663 (−1.760 to 0.434)	0.235	0.701	−0.513 (−1.613 to 0.587)	0.359	0.770
Substance use	−0.501 (−1.809 to 0.807)	0.451	0.701	0.366 (−0.895 to 1.627)	0.568	0.817
Acceptance	−0.120 (−1.093 to 0.853)	0.809	0.875	−0.224 (−1.318 to 0.871)	0.687	0.817
Suppression of competing activities	0.177 (−1.327 to 1.682)	0.816	0.875	0.040 (−1.372 to 1.453)	0.955	0.955
Planning	−0.494 (−1.984 to 0.996)	0.514	0.701	−0.172 (−1.686 to 1.342)	0.823	0.882
Abbreviations: B, unstandardized regression coefficient; CI, confidence interval; COPE, Coping Orientation to Problems Experienced; GSES, General Self-Efficacy Scale; HADS-A, Hospital Anxiety and Depression Scale–Anxiety; HADS-D, Hospital Anxiety and Depression Scale–Depression; MRS, Menopause Rating Scale.						

Values are unstandardized regression coefficients with HC3 robust 95% confidence intervals. All 15 COPE subscales were entered simultaneously. Models were additionally adjusted for nonpsychological MRS score, GSES score, age, body mass index, menopausal stage, recruitment source, education, employment status, marital status, and comorbidity. Benjamini–Hochberg false-discovery-rate correction was applied across the 15 COPE coefficients separately for HADS-A and HADS-D.
